# Power and Other Commercial Determinants of Health: An Empirical Study of the Australian Food, Alcohol, and Gambling Industries

**DOI:** 10.34172/ijhpm.2023.7723

**Published:** 2023-05-28

**Authors:** Cassandra de Lacy-Vawdon, Brian Vandenberg, Charles Livingstone

**Affiliations:** ^1^Department of Public Health, School of Psychology and Public Health, La Trobe University, Melbourne, VIC, Australia; ^2^School of Public Health and Preventive Medicine, Monash University, Melbourne, VIC, Australia; ^3^School of Social Sciences, Monash University, Melbourne, VIC, Australia

**Keywords:** Commercial Determinants of Health, Health Policy, Politics, Power, Industry Influence, Australia

## Abstract

**Background:** Commercial determinants of health (CDoH) represent a critical frame for exploring undue corporate and commercial influence over health. Power lenses are integral to understanding CDoH. Impacts of food, alcohol, and gambling industries are observable CDoH outcomes. This study aims to inform understanding of the systems and institutions of commercial and/or corporate forces working within the Australian food, alcohol, and gambling industries that influence health and well-being, including broader discourses materialised via these systems and institutions.

**Methods:** Twenty semi-structured interviews were conducted with key-informants on Australian public policy processes. Interviewees were current and former politicians, political staff members, regulators and other public servants, industry representatives, lobbyists, journalists, and researchers with expertise and experience of the Australian food, alcohol, and/ or gambling industries. Interviews sought participants’ perceptions of Australian food, alcohol, and gambling industries’ similarities and differences, power and influence, relationships, and intervention opportunities and needs.

**Results:** Strategies and tactics used by Australian food, alcohol and gambling industries are similar, and similar to those of the tobacco industry. They wield considerable soft (eg, persuasive, preference-shaping) and hard (eg, coercive, political, and legal/economic) power. Perceptions of this power differed considerably according to participants’ backgrounds. Participants framed their understanding of necessary interventions using orthodox neoliberal discourses, including limiting the role of government, emphasising education, consumer freedom, and personal choice.

**Conclusion:** Food, alcohol, and gambling industries exercise powerful influences in Australian public policy processes, affecting population health and well-being. Per Wood and colleagues’ framework, these manifest corporate, social, and ecological outcomes, and represent considerable instrumental, structural, and discursive power. We identify power as arising from discourse and material resources alike, along with relationships and complex industry networks. Addressing power is essential for reducing CDoH harms. Disrupting orthodox discourses and ideologies underpinning this should be a core focus of public health (PH) advocates and researchers alike.

## Background

**Key Messages**

**Implications for policy makers**

- Products and systems of the food, alcohol, and gambling industries have significant impacts on public health (PH) and well-being, with similarities to those of the tobacco industry.
- Powerful industries such as the food, alcohol, and gambling industries have unequalled access to policy-makers and policy-making processes. Such access is not transparent, and represents a threat to both good governance and PH.
- The current dominance of neoliberal ideological discourses is detrimental to PH and well-being. The pandemic of non-communicable diseases (NCDs) cannot be addressed through ‘business as usual’ processes.
- There are opportunities for policy-makers to play an active role in promoting PH at the policy level. There is a need to carefully reorient policy toward protecting health and well-being, and to eliminate the opaque influence of the food, gambling, and alcohol industries.

**Implications for the public**

The commercial determinants of health (CDoH) are often observable via the products and activities of specific actors and industries. The food, alcohol, and gambling industries manufacture and promote products that harm public health (PH) to varying degrees, and engage in normalising activities for their products, including at the policy and regulatory levels, in public policy processes. This research identifies the substantial power and influence of these industries, as identified by key experts. There are a number of sources to this power. However, control over framing issues, and what is and is not discussed, normalised, and therefore regulated is arguably the most dominant form of power. The finding of this study can inform public understandings of power and other CDoH in relation to the Australian food, alcohol, and gambling industries. This can be used to advocate for prioritising PH and well-being over commercial interests, and rendering political processes more transparent.

Commercial determinants of health (CDoH) are an important frame for exploring commercial influences over health. Understanding effects of health-harming products, and the industries producing them, came to prominence following revelations of tobacco-related harms.^1-3^ Today, there is concern that food, alcohol, and gambling industries use ‘Big Tobacco tactics’ and the same ‘playbook’ to produce and promote their products.^4-6^ These industries contribute to non-communicable disease (NCD) and other harm globally^5,7^ and within Australia.

 Utilising alcohol industry framings,^8,9^ food, alcohol, and gambling industries are *economic actors involved in production, distribution and marketing of food and beverages, alcohol, and gambling products or services (regardless of whether this is a primary feature of their business), as well as trade associations, and social aspects organisations.* This highlights overlaps between industries. While these industries are highly integrated in Australia, previous research has primarily examined them within research ‘silos.’

 The social, economic and political power of food, alcohol and gambling industry actors is significant. They are very active in Australia’s political environment. This is partly demonstrated by their corporate political activities (CPAs) including lobbying, political donations,^10-12^ ‘revolving doors’ moving personnel between industry and government,^13^ relationship building, and others.^14^ Previous analyses of these industries’ CPA have not generally adopted an overarching comprehensive analytical frame to guide analysis, such as CDoH.

 Examining commercial influence over political processes and governance is fundamental for CDoH analyses,^12^ given CDoH are described as arising from exertions of largely unchecked commercial power.^15^ Utilising power lenses in CDoH analyses is increasingly prominent,^16,17^ drawing on longstanding conceptualisations of power. CDoH research often focuses on what Foucault describes as *manifestation* of power^14^: the most observable qualities of power. Discourses and ideologies from which power materialises are less observable.

 Key discursive elements such as the framing of problems and possible solutions are largely shaped by powerful interests^18^ operating from specific discursive and ideological positions. These shape policy and broader social arrangements, representing an orthodoxy legitimising commercial interests, and constraining regulation, where individuals are expected to absorb risks associated with consumption. Orthodoxy, although not immutable (unlike Bourdieu’s conception of *doxa*),^19-21^ is normalised as the most acceptable order of things, largely unquestioned, and difficult to challenge. This is normalised across industry, media, and supporting structures to shape the views of the general population, regulators, and politicians alike.

 Prominent CDoH framings describe commercial activity promoting products and choices detrimental to health^22^ and health influences stemming from the “profit motive.”^23^ CDoH scholarship now increasingly recognises CDoH complexity, whereby outcomes include health harms and/or benefits, sometimes concurrently.^24,25^ In this paper, CDoH are framed as *system(s) of commercial and/or corporate forces that have potential to influence health and well-being*, including systems of power, and political and economic ideologies and discourses reinforcing these. This includes discursive and other systems of neoliberal capitalism that have mostly succeeded in advancing commercial interests above public health (PH).^26^ While power is often described as integral to investigating CDoH, CDoH power framings have largely been conceptual rather than empirical.

 Some recent CDoH analyses used Lukes’ *Three Faces of Power,*^17^ or Fuchs’ adaptation of Lukes’ work: *Three Forms of Corporate Power.*^16,27^ Wood et al. compiled an *Integrated Corporate Power Framework to inform Analysis of the CDoH*.^16^ This draws on Foucault,^28^ Fuchs,^27,29^ Fuchs and Glaab,^30^ and Madureira-Lima and Galea,^31^ describing corporate power as having material (ie, ownership, financial means, information, technology) and ideational (ie, knowledge, perceived legitimacy, ideas, values, norms, etc) origins; and instrumental, structural, or discursive natures; which manifest in corporate, social, and ecological outcomes. This framework is used to frame the discussion in this study.

 Many CDoH theorisations are categorical rather than explanatory. These describe types of power pertinent to CDoH, categorising power relations without describing processes that materialise these. Understanding of the underlying qualities of this power, permitting the less visible elements of power to be identified or mapped in practical terms, is rare.

 This study brings a power lens to an empirical analysis of CDoH within Australia. CDoH systems are observed via the reporting of policy-making, regulatory, CPA, relational and other processes by those familiar with them. This work aims to understand the systems and institutions of commercial and/or corporate forces’ potential influence over health and well-being pertaining to the Australian food, alcohol, and gambling industries. This includes identifying and moving towards an understanding of the health and broader discourses materialised via these forces and institutions.

## Methods

 Semi-structured interviews were conducted with key-informants with experience working or liaising with, or as representatives of, Australian food, alcohol, and/or gambling industries. This included current and former politicians, political staffers, regulators and other public servants, industry representatives, lobbyists, journalists, and researchers. Individuals with experience across multiple sectors were particularly sought for their breadth of experience.

 Key-informants were contacted via publicly accessible contact information, authors’ networks, or snowball sampling. Contact lists were developed per the Sample Frame (Supplementary file 1). Email invitations were sent, followed by up to two weekly reminders. Where individual email addresses were unavailable, enquires were submitted via organisational addresses or online contact portals. If these were unavailable, invitees were phoned. Explanatory statements were sent approximately one week, or as early as possible, before each scheduled interview. Before each interview, the interviewer confirmed participants had received the explanatory statement and addressed any questions. Informed consent was confirmed verbally before recording commenced. Participation was voluntary with no remuneration offered.

 The first author conducted interviews between January and March 2021 via phone or Zoom teleconferencing. Interviews took 30-60 minutes, were audio recorded, blinded, and transcribed by a professional transcription service. The interviewer also took detailed notes.

 Participants were asked their perceptions of the Australian food, alcohol, and gambling industries, including similarities and differences, power and influence (including soft and hard power), industry relationships with government, advisory, lobbying, union, consultation, and other roles, regulatory and other mechanisms reinforcing influence or power, and opportunities, needs and priorities for interventions to reduce harms and enhance benefits. Hard power was conceptualised as coercive power (eg, legal and/or economic threats, use of force), while soft power was conceptualised as persuasive power (eg, shaping preferences, influencing through perceived legitimate or moral authority).^32^ The interview guide (Supplementary file 2) was informed by a recent CDoH systematic review.^33^

 Data were analysed thematically. The first author coded transcripts thematically utilising NVivo software. The coding frame was developed reflexively in multiple stages in consultation with all authors. This was initially based on the interview guide, then revised based on participants’ responses. Following this, themes emphasising neoliberal discourses became prominent, including ‘choice-centric’ themes emphasising individual agency, voluntarism and choice, advocacy for limited state intervention, and perceptions that state intervention limits individual agency.^26^ Alignment of these themes with prominent orthodox neoliberal discourses is highlighted throughout.

## Results

 Sixty-four people were invited to participate. Forty (62.5%) responded, with 28 expressing interest. However, four could not interview within the recruitment period, one felt they lacked required expertise, and one responded after recruitment concluded. Two industry representatives who scheduled interviews withdrew before completing these due to perceived conflicts of interest with their work.

 Twelve individuals declined invitations, with four referring colleagues. Reasons for declining included disinterest (n = 4 industry representatives) and work commitments (n = 2 journalists). Four regulatory bodies and a food industry body said nobody was available and/or that all relevant information is already on public record. One gambling industry body emphasised no representatives would participate, complaining to our faculty about perceived researcher bias from alleged ‘anti-gambling’ sentiments.

 Twenty interviews (31.25% of invitees) were conducted (7 women; 13 men), via Zoom (n = 11) or phone (n = 9). Participants represented jurisdictions across Australia (Australian Capital Territory, New South Wales, Northern Territory, Queensland, South Australia, Tasmania, Victoria, and Western Australia), with experience and expertise in the food (n = 12), alcohol (n = 15), and gambling (n = 9) industries. All key-informant groups were represented (Table). Most had experience and expertise across multiple areas. The four overarching themes are discussed below.

**Table:** Participant Details

	**Invited to Participate**	**Interested in Participating**	**Not Interested in Participating**	**No Response Received**	**Interview Scheduled**^a^	**Interview Completed**^b^
Politician	4	4	0	0	3	3
Political staffer or advisor	7	5	0	2	5	5
Regulator	12	5	3	4	5	4
Lobbyist^c^	6	3	0	3	3	3
Industry representative	31	9	9	13	8	6
Journalist	11	7	2	2	3	3
Researcher	8	7	0	1	6	5
Food industry	29	17	4	8	13	12
Alcohol industry	35	16	6	13	16	15
Gambling industry	33	15	7	11	9	9
**Total**	**64**	**28**	**12**	**24**	**22**	**20**

Numbers may total more than column totals due to participants’ experience in multiple areas. ^a^Four unavailable due to leave or scheduling issues; one was not confident of expertise in Australian context; one responded after recruitment completed. ^b^Two industry representatives cancelled their interviews on the day they were scheduled to be conducted due to concerns about conflicts of interest with their work. ^c^Australia has a relatively narrow definition of lobbyists: “any person, company or organisation that conducts lobbying activities on behalf of a third party client or whose employees, contractors or persons otherwise engaged by the person, company or organisation conduct lobbying activities on behalf of a third party client.”^34^ There are some exclusions for charities, member-based not-for-profit organisations, trade delegates, professional members, and some others. Lobbyists must be registered, and Australian Government representatives can only meet with registered lobbyists. It is important to note that not all undertaking lobbying activities (including employees of corporations, for example) are considered lobbyists under Australian legislation.

###  Industry Similarities and Differentiation

 Participants identified regulatory, structural, and behavioural similarities between Australian food, alcohol, and gambling industries. These included industry integration via organisational structures (eg, where industry bodies represent multiple industries), supply chains and logistics (eg, around fast-moving consumer goods), and regulatory structures (ie, where regulatory bodies regulate multiple industries). Industries were described as employing similar strategies from the same ‘playbook.’

 “*… the same key peak bodies can cover different industry sectors, food, soft drinks and alcohol, hotels, gambling … It seems to me that there is a very similar playbook … working in their own best interests through lobbying politicians through political donations either directly or indirectly*” (P2, researcher).

 Participants also differentiated between industries. Almost all participants compared food, alcohol, and/or gambling industries with the tobacco industry. Most highlighted differences between these (ie, saying these industries’ products are less harmful than tobacco). However, some PH-focussed participants emphasised similarities between these industries’ harms and/or tactics. Participants implied a hierarchy between industries based on perceived ‘need,’ utility, and harm, where food is least harmful and most necessary, while tobacco is most harmful and least necessary (see Figure).

**Figure:**
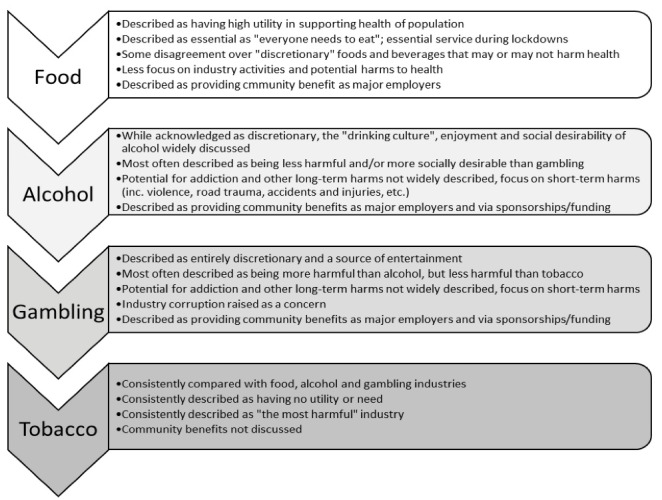
The Hierarchical Need, Utility and Harm Associated With the Australian Food, Alcohol, and Gambling Industries.

 “*Food we actually need and so that’s a much more complex … they get more and more complex in that hierarchy … from tobacco through alcohol to food and probably gambling is closer to tobacco*” (P4, politician, researcher).

###  Perceptions of the Existence and Nature of Power and Influence 

 Participants reported mixed perceptions of the extent of food, alcohol, and gambling industries’ power. Industry participants, and some with political experience, downplayed the influence of this power over policy decisions. Researchers, journalists, regulators, and some others took a contrary view.

####  Hard Power

 As for power overall, industry participants and some political participants disputed the exertion of hard power over political decisions or processes.

 “*I don’t think that there’s … coercive power because at the end of the day advisers are people, they’re going to make their best judgment to benefit obviously their Minister, and it’s just politically stupid if anyone favoured one group over the other*” (P18, political staffer, lobbyist, industry representative).

 PH-focussed participants instead described many CPAs as exertions of hard power, including financial power, economic threats and legal threats.

 “*Talking about monopolies and I guess oligopolies is I think that’s another power that food and probably alcohol companies have … almost by the size of them shift any other competitors out of the market*” (P10, researcher).

 Participants discussed industries making legal threats against individual politicians, political staffers, journalists, news outlets, and would-be industry whistle-blowers. These were particularly discussed for the gambling industry.

 Significant financial resources were said to allow industry to readily take legal action, including fighting policy reform.

 “*Money is a big one, so they’ve got the money to legally fight reform. If you think of tobacco for example, the money they spent on fighting plain packaging. Or if you think of the food industry, the money and resources they allocate towards fighting more clear packaging and labelling of their products. Alcohol, the amount of money they spend on trying to stop pregnancy labels from being placed on their products. They will invest in legal teams to do that*” (P12, journalist).

 Economic power was said to arise from corporations’ size, market power, and revenues, which frequently exceed those of national economies. Economic threats in response to policy reform were widely described by PH-focussed participants. Threats of job losses, and corporations shifting operations offshore (with anticipated job losses and decreased tax revenues), were highlighted. Some emphasised that industry often overstates these.

 “*[Their economic power is] why there is so much power to thwart governments because there’ll be the power to withdraw the operations to offshore … all a corporation needs to do is say well we’ll take our bat and ball and we’ll go somewhere else and there’s capitulation from governments*” (P2, researcher).

 Significant financial resources were said to make corporations resilient, facilitate media buying, including large-scale advertising to shape public narratives, and facilitate political donations. Commercial entities’ rights, such as corporate personhood rights, or rights afforded under trade agreements, were also described as exertions of hard power.

####  Soft Power

 While almost all participants alluded to soft power, perceptions of influence again differed by participants’ experience. Power to shape narratives and frame issues was most commonly described. These often related to reinforcing cultures normalising alcohol and gambling products, and occasionally ultra-processed foods.

 Alcohol and gambling were described as part of Australia’s cultural history, originating in the colonisation Australia. Modern industry narratives promoting products as ‘fun’ and ‘social’ were described as pervasive and ignoring health concerns.

 “*I don’t think they’re promoting alcohol abuse by any means, but they are certainly creating a culture and it’s a culture of ‘gambling is fun and just part of this experience’ [and] is sort of definitely quite pervasive” *(P19, regulator).

 Most participants agreed Australia has a drinking and gambling culture. Within this, participants implied there is a ‘right’ culture of moderation, while cultures of excess should be discouraged. Culture was often described in connection with underlying narratives of individual responsibility, where individuals were (rather contradictorily) expected to ignore normalising social, cultural, marketing and other influences to make informed, ‘healthy,’ ‘responsible’ choices.

 Some discussed soft power as perpetuating broader neoliberal capitalist discourses, including roles of government, business, and consumers.

###  Perceptions of Sources of Power

 Participants described sources of power including relationships, social responsibility, and others.

####  Relationships

 Power was widely described as arising from close relationships between industry and government. Facilitators of these reportedly include advisory roles, committee memberships, revolving doors between industry and government, formal lobbying activities, political donations, gifts, and other CPAs.

 All participants discussed lobbying, communication, consultation, and/or stakeholder management between industry and government, making lobbying the most described CPA. Even participants who disputed industry power emphasised government relationships as important for prosperity. One industry participant discussed not undertaking lobbying activities, before emphasising the ease of forging government relationships:

 “*I think anyone, if they actually take the time and effort, can build a relationship with a ministerial adviser... If you wanted to go and form a relationship with a ministerial adviser you’ve just got to go up and make your case and ask, and I honestly don’t think I’m being naïve in that...”* (P8, political staffer, industry representative).

 Political donations were commonly described as facilitating relationships between industry and government. PH-focussed participants described the political donations system as “a real mess” (P12, journalist) and lacking transparency. Some went further, describing donations and gifts as exerting influence over politicians, constituting corruption.

 “*Well the donations thing is very complicated and the money in itself doesn’t explain everything… it’s about the whole political system really… but, if you ban donations or you reduce them to the level that they’re relatively unimportant then you do take a lot of pressure off politicians, therefore off bureaucrats and so on*” (P9, researcher).

 Some participants, primarily industry representatives, said access to politicians afforded by political donations has been overstated, while discussing the ease of accessing politicians via other means.

 ‘Revolving doors’ were described as fostering close working relationships between industry and government. This was positively described by industry participants and some political participants, but negatively from PH-focussed participants. This was also true for views on industry participation in advisory committees, and industry influence over science.

 Relationships between industry and government were described as being fostered within formal and informal settings (including social settings) alike. One participant described relationships as “the single biggest mechanism” of power:

 “*… the kinds [of relationships] that are really influential are not the formal public ones. They’re the things that happen in private quite often. In Boardrooms, in Airport Lounges, in Parliament too… And there’s just no comparison in terms of the amount of contact… how can you help but be influenced as a human?”* (P12, researcher).

###  Corruption

 Corruption was described as the power to prevent warranted policy development or implementation, and ‘turning a blind eye’ to malpractice. This was seen to result from excessively close, influential relationships between industry and government.

 “*You know, there is no political problem in the world that doesn’t exist except for the fact that somebody is making money out of it. Everything else can be fixed. Everything would get fixed tomorrow except someone’s making money and somebody then passes that money on to somebody with enough power to either do the right thing or prevent the right thing”* (P1, political adviser, journalist).

 Corruption was seen as concerning the integrity of elected officials, policy systems, and regulatory bodies alike.

 “*It’s quite alarming how so many agencies at state and federal levels have failed us in this [corruption]”* (P7, politician).

 This framing is broader than legal definitions of corruption. One participant discussed this further:

 “*You know, what we need at a federal level is a very broad definition of corruption. But not just criminal corruption. But you know, the definition of what is seriously improper conduct. What is corruption? That may not be a criminal offence but it’s wrong and someone should be held to account”* (P7, politician).

####  Social Responsibility 

 Social responsibility was a prominent theme. Participants described industry as ‘socially responsible’ and ‘part of the solution,’ and corporate social responsibility (CSR) activities as important for maintaining industries’ ‘social licence to operate.’ Some said this brings significant industry power.

 Activities described included providing employment (particularly for vulnerable groups), financially supporting community organisations, and providing enjoyment and leisure. Some participants emphasised that this involves industry downplaying the extent of the ‘problem’ and their contribution to it, and/or reframing the problem on their terms.

 “*[industry are] using the statistics [about] how good it is for the community and what goes back to the community, how many jobs there are. But they don’t count or put a dollar amount on the destruction that’s caused by gambling, alcohol. You know, we’ve got people with gambling addictions that are hospitalised, suicides – 400 suicides a year – none of that is taken into account” *(P11,industry representative).

 Overall, individual consumer ‘responsibility’ and consumer choice were emphasised. Food and alcohol industry representatives particularly denied or downplayed links between consumption and NCDs. Discussions of product safety focused on the mitigation of short-term harms (ie, communicable disease outbreaks or sessional harms). For gambling, this included emphasising the ‘small’ proportion of ‘problem gamblers,’ or an individuals’ ‘problem.’

 “*I think the system, that’s the policy and the regulatory system is very much framed in terms of more immediate acute impacts on health. And we don’t tend to look at the far more important and significant areas from a health and economic perspective of poor sustainability, what it’s doing to undermine the ecological basis to the food system as well as the chronic diseases and so on that come along”* (P20, regulator, researcher).

 Some participants described social and financial benefit to industry from promoting CSR activities. These CSR activities were at times described as strategically designed to avoid further industry regulation or government intervention.

 “*You can make the judgment that you should probably assume that half of this is actually motivated by good corporate social responsibility and half of it is motivated by doing it yourself and being seen to do it so that the government doesn’t step in and do it in a much more heavy-handed way” *(P3, political staffer, lobbyist, industry representative).

####  Other Sources of Power

 Other sources of power included overblown claims of economic importance, including providing jobs important for the national economy. However, some participants criticised these claims:

 “*So and they use the normal you know ‘It’ll cost jobs. It’ll hurt. It won’t work,’ all the slogans. Yeah which there’s no truth in that*” (P11, industry representative).

 Some participants emphasised the role of media in shaping public and political opinions, and described the extent to which industry utilises this:

 “*I think a lot of people don’t realise how influential the media is and its ability to change public opinion*” (P14, lobbyist, industry representative).

###  Needs and Opportunities for Intervention 

 Needs for intervention were framed primarily through neoliberal ideologies, with varying support for government intervention. Similarly, the importance of individual freedom and choice were emphasised to a greater or lesser extent. Views expressed differed notably based on positions participants had held (eg, researchers compared to industry representatives), and, for those with political experience, political orientation.

####  Role of Governments

 Participants with industry, lobbying, and/or conservative government experience, including politicians and political staff, more commonly advocated for ‘hands-off’ government approaches and industry self-regulation. They instead indicated that consumers should take responsibility for their own health.

 “*But the government can only do so much and I think a lot of people put a lot of emphasis that ‘Oh, I have a problem, the government should fix it,’ and it’s like ‘Well no, you have two arms and two legs, you fix it yourself’”* (P18, political staffer, lobbyist, industry representative).

 PH-focussed participants were more in favour of regulatory intervention.

 “*These problems are not unique to alcohol and tobacco … they’re suffering the same problem of people’s private profit motives. There’s nothing wrong with a profit motive, what’s wrong is failing to regulate it. So I get a bit annoyed when I hear people saying well industry needs to do better. It’s not industry, it’s government needs to do better in regulating industry … this idea of self-regulation … is just utterly ridiculous” (P12, journalist).*

 Industry representatives more often asserted that current regulation is adequate. Some PH-focussed participants indicated that industry reluctance for further regulation arises from wanting to maintain profits.

 “*The more effective you are at minimising harm, the more you reduce gambling addiction and that wipes out the profit … But I say to businesses that if you’re relying on gambling addiction, you shouldn’t be in business” *(P7, politician).

 All participant groups discussed value in ‘working relationships’ with government. Industry members favoured ‘all working together’ where government, industry, and PH work together to develop effective policy and practice. Conversely, PH-focussed participants advocated against this approach, saying industry uses these relationships to co-opt agendas and promote industry-favoured outcomes. Instead, PH-focussed participants recommended restricting industry access to government personnel and processes.

 Perceptions of necessary reforms also differed by perspective. PH personnel, regulators, and journalists alike advocated for increased regulation. These participants emphasised more closely regulating relationships between industry and government, particularly for political donations, lobbying, revolving doors, and corruption. These participants described the need for greater emphasis on integrity, and the need to pass the ‘pub test’ (ie, to be perceived as reasonable conduct by ordinary Australians).

 Addressing corruption was a key priority for these participants, although not all used this terminology. Those who did argued that Australia’s narrow legal definition of corruption should be expanded to better promote the integrity of political and policy systems. Others advocated for a federal anti-corruption watchdog with enforcement abilities.

####  Role of Education

 Participants from all groups emphasised needs for additional public education to minimise harms and/or promote health. This included educating consumers and politicians alike. Participants generally described the need for broad education campaigns (eg, food labelling interventions, alcohol pregnancy warning labels, etc).

 Public education needs were often described within broader individual responsibility narratives. Industry representatives, and some political participants, emphasised the need to inform consumers about products so they can make good health choices. Some participants discussed needing to increase politicians’ and political staffers’ health literacy. PH-focussed participants more often advocated for educating consumers on industry activities, and how to protect themselves from these.

####  Freedom and Choice 

 Arguments about interventions, government roles, and overall responsibility for harms caused were also underpinned by freedom and choice arguments. Participants cited ‘consumer sovereignty,’ an important element of neoliberal economic discourses.^35^ Underpinning this is the idea that consumers should be free to make choices, and that government should educate, and therefore empower consumers with knowledge to make the ‘best’ decisions for themselves.

 Some PH-focussed participants emphasised that consumers rarely make truly informed choices. Factors discussed as restricting individual agency included commercial ‘nudging’ and ‘choice architecture’ tactics; omnipresent marketing; and markets flooded with unnecessary and/or unhealthy products.

 While addictive properties of alcohol and gambling products were raised by approximately half the participants, effects of addiction on capacity for rational and informed choices and behaviours were rarely emphasised. Instead, a few participants indicated that even those experiencing addiction are responsible for their behaviour.

 “*I know that there’s problems [with gambling] and people have issues – that goes across any addiction – but, a lot of that money floats back into the regions … I wouldn’t call it a necessary evil but it’s a humans’ choice and it’s like anything, anyone can have an addiction, even an addiction to food. But do I think that there should be that kind of stringent regulation on food, alcohol? No, because it’s a legal substance”* (P18, political staffer, lobbyist, industry representative).

 Some PH-focused participants emphasised that people experiencing addiction need additional help managing behaviours and mitigating harm.

 “*Like any kind of addiction, if you have a gambling addiction, then it’s very hard to control and to manage on your own. So we need to help those people. So we have all these rules around what venues can’t do*” (P6, regulator).

 Two PH-focussed participants linked arguments about choice with health first paternalism.

 “*The right to freedom and liberty and the right to choose may be a detrimental path but live with the consequences. So health-first paternalism is a way of saying ‘leave me to make my choices, get out of the public health domain and give us all our freedom’*” (P2, researcher).

 Another described the ‘freedom’ that industry advocates for as another form of domination.

 “*Industry always comes back to ‘we should be free’ and what they want is freedom from interference … that’s not freedom, freedom is about domination. When you frame things in terms of domination what the tobacco industry, the alcohol industry, the food industry wants to do is to maintain their domination … So that’s why Governments not only have a role to regulate they actually have a responsibility to regulate in this area and they’ve failed in that responsibility with regard to junk food. They have failed in that responsibility largely with regards to alcohol and they’ve failed in that responsibility with regard to gambling”* (P4, politician, researcher).

## Discussion

 This study examines aspects of the power of Australian food, alcohol, and gambling industries within a CDoH frame. This includes social, political, regulatory, and other mechanisms, and the influences these exert on public policy and PH. Food, alcohol and gambling industries significantly affect the health and well-being of Australians. Together, industries’ power shapes consumer’s relevant decision-making environments,^36-39^ and important health, social, and political discourses.^18,40-43^

 The following sections discuss the findings of this study in the context of Wood and colleagues’ framework.^16^

###  Manifestations of Corporate Power 

 CDoH literature has arisen largely from concerns about *manifestations* of power^28^: the most visible forms of power. Participants primarily described manifestations of corporate power as negative (ie, as harms). Industry participants more often suggested positive or neutral outcomes.

 Participants largely focused on social outcomes. While PH-focussed participants described health, social, political system, and other harms, industry-focussed participants touted societal benefits. These centred on providing valued (and sometimes essential) goods and services, and social and economic benefits via jobs, economic participation, and similar. This is consistent with previous analyses of industry discourses^2-6^ and to be expected based on participants’ perspectives and interests.

 Corporate outcomes, such as profit maximisation, were primarily described as negative (eg, profiting from unethical practices). This is consistent with broader CDoH literature.^22,23,44,45^ Only a few PH-focused participants described ecological implications of unsustainable food systems and broader unsustainable production systems. This is consistent with short-term framings of harms noted here and more broadly as discussed further below.

###  Nature of Corporate Power

 Fuchs’ *Three Forms of Corporate Power* can be used to describe the different natures of corporate power discussed above.^27^

####  Instrumental Power 

 Instrumental power includes political and policy influences arising from mechanisms like lobbying and political donations, also described as CPA.^46-47^ Participants, and particularly PH-focussed participants indicated that Australian food, alcohol, and gambling industries wield considerable instrumental power. Industry and political participants widely described CPAs, while denying their influence.

 Regardless of whether CPAs ‘convert’ into policy favouring industry, industry actors’ unequalled access to politicians and their staff demonstrates significant instrumental power. However, the casual language used by industry and political participants downplays this. These participants’ views reflect orthodox discourses normalising industry relationships with policy-makers as natural and essential. Concepts of political corruption, framed narrowly and in a strict legal sense, similarly reinforce these orthodox discourses, shifting focus from broader integrity issues including acting with honesty and morality. Corporate influence relies on abilities to influence the institutions of government, arguably creating less democratic, more plutocratic institutions,^48,49^ exerting disproportionate corporate power, and prioritising commercial interests above PH.

 Many participants downplayed or denied the influence of relationships and other power dynamics. Logic dictates these instrumental power mechanisms would have limited investment if they were truly inconsequential. Instead, these mechanisms are evident across multiple, intentional activities. For instance, dark money donations, or donations where the source is not published, are often dismissed as inconsequential,^50,51^ as also noted in this study. If indeed unimportant, transparency would not be an issue. However, available evidence suggests these and other CPAs are indeed globally powerful.^6,10,11,13,14,50,51^ This influence distorts the institutions of government and regulation.

 Mechanisms for disrupting instrumental power were priorities for PH-focussed participants, including some with political experience, regulators, and journalists. The interventions discussed focused on reducing CPAs and relationships between industry and government, and therefore increasing integrity in policy-making and politics. These approaches, including restrictions on lobbying, political donations, revolving doors, gifts, and others, with proper enforcement of these, have been discussed widely in the CDoH literature.^45,52,53,54,55,56^ However, Australia has relatively weak requirements for transparency in these areas, and weak enforcement of existing regulation.^57,58^ The lack of transparency and the lack of consequence for those breaching or noncompliant with regulation in these areas is a prominent barrier to reducing the exertion of instrumental power in these areas. Legislation for a new Australian National Anti-Corruption Commission passed the parliament in November 2022.^59^ However, the effects of this remain to be seen.

 Participants also placed some emphasis on upskilling the public and officials in recognising and recognising the effects of CPA as a means to delegitimise these tactics and reducing instrumental power. There has been emphasis on monitoring and exposing corporate activities in recent literature as a means counter the corporate ‘playbook,’^46,53,56,57,60,61^ with a range of organisations undertaking corporate watch activities. This work is essential to counter the orthodox discourses that reinforce CPA as essential or normal, as are research translation activities around this to make this work accessible in the policy and broader public environments. Further, continuing pressure needs to be applied to achieve real-time disclosure of political donations, reduce the threshold for disclosure of such donations (in Australia it is currently more than $14500),^62^ and make parliamentarians’ diaries available for public scrutiny. Certain types of donors are prohibited from donating to political parties in one state, including property developers and commercial gambling operators,^63^ but no such restrictions exist at the national level.

 Meanwhile industry and political participants, denying the influence of instrumental power, did not suggest any need to address this. This is entirely in line with previous observations about industry’s use of instrumental power and the prominent industry discourses.^2,3,52,53,56^

####  Structural Power 

 Structural power allows political agenda-setting and shaping markets and other environments, using institutional processes involving private investment, employment, taxation, economic activities, legal action, and others.^27^ This power derives from commercial and economic capital accrued via reflexive processes. Entities exercising this power promote and benefit significantly from favourable policies. Participants widely described structural power when discussing hard power. While some industry representatives disputed the use of hard power, others described industries’ economic threats to withdraw from Australia to avoid reforms, shift competitors out of the market, and emphasise their economic importance within Australia. Some described industries’ use of legal structures including trade agreements and broader legal structures to pursue business goals and avoid ‘restrictive’ regulation.

 Examples of this structural power have also been described elsewhere. Trade agreements between the United States, Mexico, and Canada reportedly favour industry by expanding intellectual property rights that will increase pharmaceutical costs and weaken health and food safety oversight.^64^ Analyses of McDonald’s Australia reported that while they emphasise offering significant employment opportunities, these are predominantly for younger people, lower wages, and insecure employment contracts.^65^ Meanwhile, tobacco industry legal challenges to plain packaging policies in Australia, the United Kingdom, Canada, and the Netherlands presents another example.^66^

 Structural power may be a consequence of access to power via resources including capital, control of orthodox discourses, and access to politicians and political processes. These appear ‘structural’ because they produce material effects, and are enhanced by processes increasingly reliant on commercial actors’ resources and inputs.^27^ Ongoing privatisation of goods and services that have traditionally been government responsibilities^26^ may likewise contribute to these. Further, globalisation has expanded the market dominance of ultra-processed food, tobacco, alcohol, and other products, deriving significant private profits, while contributing substantially to population harms, and therefore public costs.^26^ Meanwhile, increased privatisation of public systems enhance private profits^26^ These effects arise from orthodox discourses promoting the value and necessity of privatisation and globalisation, facilitating what can be observed as structural power.

 Structural power cannot be examined in isolation, and particularly not in isolation from discursive power.^67^ There are several examples of the reduction of structural power. This has included using structures like financial power and disinvestment practices. Perhaps the most prominent of these are in tobacco and arms. Tobacco Free Portfolios have worked to divest from tobacco in some of the world’s largest financial markets including banking, insurance, pension funds, wealth management, and others.^68^ Similarly, Quit Nukes and Don’t Bank on the Bomb work for divestment from nuclear weapons among financial institutions and have seen good success.^69,70^ However, these shifts have happened in line with international shifts in the legal environments (ie, the Framework Convention on Tobacco Control and the Treaty on the Prohibition of Nuclear Weapons)^71,72^ and broad societal shifts in discursive power surrounding the tobacco and arms industries in particular.^73^ Therefore, approaches to addressing structural power need to be considered in conjunction with discursive power.

####  Discursive Power 

 Discursive power was highly observable in this study. That is, the power to pursue and shape interests by shaping societal values, norms, and ideas.^27^ This is observable via participants adopting and promoting neoliberal concepts, discourses of industry self-and light-touch regulation, and emphases on individual responsibility and freedom of choice. These discourses fundamentally shape relationships between populations, industries, and government,^26,41,74^ and the distribution of power.

 Varying participant perceptions were expected, given that experiences and subjectivities shape perspectives.^75^ Industry and some political participants’ narrow framing of ‘health,’ focussing on short-term over long-term or cumulative outcomes, and individuals over populations, is consistent with regulatory approaches focussing on short- rather than long-term harms, and orthodox discourses underpinning these. Meanwhile, PH perspectives of harms and health represent generally heterodox discourses opposing this orthodoxy.^20^ The ‘lifestyle drift’ phenomenon may partly explain participants’ tendency to describe upstream determinants of health, including CDoH, and then propose interventions targeting more proximal determinants (eg, education).^76^ This suggests it is challenging for participants to articulate interventions transcending orthodox discourses.

 Orthodox discourses perpetuate and reiterate the ‘reasonableness’ of industry actions, legitimising industry’s inclusion in regulatory and decision-making processes, by emphasising industry benignity and neutrality.^76^ Meanwhile, those questioning the usefulness and/or conflicts of interest involved in these are portrayed as ‘unreasonable.’ Orthodoxy pursues its own logic, adhering to, and reinforcing, orthodox power.^20^ These powerful actors thus shape what is and is not deemed reasonable. Within this, choice and individual responsibility are often prioritised above protecting people from harms. Individual responsibility discourses manifest as promoting ‘drinking responsibly’^77,78^ or ‘gambling responsibly,’^40,41,79^ emphasising personal or parental responsibility for diet.^80,81^ In these, products are portrayed as benign, while the industries producing, promoting, and distributing these are portrayed as neutral actors promoting consumer autonomy, enjoyment, and choice.^41,82^ While participants conceded that alcohol and gambling were probably not essential, food was disputed. Some emphasised that not all food products are essential, and once long-term harms are considered, the harms arising are often significant. This claim is supported by previous evidence.^5,83,84^

 Orthodox discourse likewise constructs image and harm. Participants frequently framed ‘social drinking’ or ‘social gambling’ as ‘good’ and ‘responsible’ behaviours, while drinking or gambling alone, or to ‘excess,’ are ‘bad’ and ‘irresponsible.’ Participants also made arbitrary distinctions between types of alcohol and consumption settings, reinforcing discourses, such as individual responsibility discourses, regularly articulated by industry. These framings are, strategically, frequently at odds with true harm. For instance, there is good evidence of long-term harms from regular alcohol consumption,^85-87^ regardless of setting or type of alcohol consumed. This is consistent with the broader pattern of industry discourses that insist there is no problem or admit there is a problem but insist it is less severe than other problems or less severe than people assert.^3^ This works to obfuscate the issue and generate doubt. The generation of doubt is, of course, a tobacco industry tactic adopted by others.

 While participants were not asked about the tobacco industry, almost half discussed this. The implied hierarchy this reveals with tobacco as most harmful, followed by gambling and alcohol, with food the least harmful, is consistent with previous research^33^ and entirely consistent with industry discourses. While this hierarchy may truly exist, it is unlikely that the differences from tobacco are as stark as implied by some participants. Some authors describe this hierarchy as giving some industries unfair advantage to promote their products and avoid regulation.^23,33^ Unsurprisingly, food, alcohol, and gambling industries reportedly adopt these comparisons to avoid the regulation placed on tobacco.^1,4,6,88^ However, PH evidence generally favours further regulation.^5,89-92^

 Australia has seen successes in implementing relatively strict tobacco control, including tobacco plain packaging laws introduced in 2012.^93^ These laws are the latest in Australia’s tobacco control policies implemented incrementally since the 1970s^94^ which have been quite successful. Despite multiple legal challenges, the tobacco industry has been unsuccessful in overturning these laws.^93^ These tobacco control policies have gradually decreased the capacity of the tobacco industry to promote their products and interests in the public domain. Meanwhile, the revelation of documents from within the tobacco industry detailing the industry’s tactics and increasing awareness of the true harms caused by tobacco likely contributed to a ‘de-normalisation’ of tobacco: increasing scrutiny, highlighting bad behaviour, and decreasing contact to policy-makers for the tobacco industry.^95^

 However, the tobacco control approaches taken within Australian and global contexts has fed a discourse of tobacco exceptionalism – where tobacco is portrayed as a product unlike any other, presenting a unique threat to health, and therefore warranting special regulation while other products causing similar harm do not.^96,97^ Tobacco exceptionalism plays well into other industries’ discourses and is not unique to Australia.^98^ Meanwhile, the example that has been made of tobacco has served as a warning and a learning opportunity for other industries who do not want to suffer the same fate of becoming ‘persona non grata.’^5^

 Together, these have at least in part contributed to Australia’s regulatory failure in a range of other policy areas including the failed carbon pricing scheme and the failed introduction of gambling machine pre-commitment. In both these instances, the respective industries were highly effective at mobilising against further regulation, controlling discourses, drawing on personnel and significant financial and other resources, acting aggressively, and increasing efforts to maintain their collaborative position with government.^99,100^ Similarly, taxing sugar-sweetened beverages has been proposed several times but has never seriously been on the policy table. Because these products are ‘not as bad as tobacco,’ the relationships between the products and the harms are arguably more complex, and PH responses have not always been clear or singularly focused, these industries have fostered sufficient doubt to maintain policy inaction. The discursive lessons in this are that tobacco should not be the benchmark against which all harms are measured. It is important for PH actors to improve communication of complex causal relationships. Relevant policy advocacy efforts need to be clear and consistent.

###  Origins of Corporate Power 

 Fuchs and Glaab describe two sources of corporate power.^30^ Material power originates in economic capabilities and is used to influence political processes’ inputs and outputs.^30^ Financial means enable many CPAs, inducing financial dependence on corporate actors amongst political decision-makers and others.^30^ Other interests are often unable to match this, while corporate actors further expand their financial resources as a result of political activities.^30^ Participants prominently identified material origins of power. How material resources translate into political influence are important considerations.

 Ideational power includes framing political issues, shaping discourse surrounding policy definitions, actors, norms, and processes,^30^ and defining what is *not* on the political agenda. This power constrains or engrains behaviour and action by controlling symbolic meanings within social practices and institutions.^30^ Knowledge, and how knowledge is processed and interpreted, is key to this.^30^ Ideational power is the ability to shape orthodox discourses, to determine what is meaningful or normal, and generate self-perpetuating logic. Many participants discussed this.

 Participants also described relationships as a prominent source of power. While some relationships may be ideational (ie, shared world-views, aligned agendas), and some material (ie, similar material power), some relational power likely lies outside the material-ideational binary. Commercial actors have power. However, some individuals also articulate power. Often, these people are sought for the power (connections, contacts, and orthodox ideas) they bring to industry and other roles. Thus, the appeal of revolving doors between government and industry.^13^ For corporate power, it is important to acknowledge relevant orthodox power possessed by individuals who know how to identify, articulate, and deploy power.

 Another origin of power some participants touched on is the interconnection between food, alcohol, and gambling industries and other related industries including media. Organisations like trade associations, advertising agencies, public relations firms, consulting firms, corporate law firms, financial firms, major retailers, logistics and warehousing companies, and some trade unions also have co-dependent relationships with these industries. In this, food, alcohol, and gambling industries’ power is bolstered by powerful and often mutually supportive industry networks. Aligned interests between industries and associated actors translate into generally aligned discursive, structural, and instrumental power, meaning policy advocacy efforts are likely to be successful.

 In this study, participants emphasised that power provides access to policy-makers, and political processes, while influence is the ability to use or convert power into action. This aligns with previous power and influence framings.^30,101^ However this conversion is not always direct. Often, influence is exerted by shaping orthodox discourses and therefore shaping what is viewed as problematic, reasonable, and otherwise. This is highly reflexive as power begets and embeds power constantly. That is not to say that orthodox power is insurmountable, but that it dominates social and political spaces.

###  Implications for Public Health

 Power cannot be ignored when analysing PH challenges arising from food, alcohol, gambling, or other industries. These products have significant health implications, and these industries have considerable power over social, commercial and political environments, consistent with orthodox discourses. Prevention and harm reduction efforts cannot succeed without addressing this power.

 Determinants of health, and especially CDoH, are constituted via complex orthodox systems, underpinned by supportive discourses and ideologies. Opposing or amending these systems to better serve PH requires adopting a heterodox position, and proposing interventions to amend each element of the orthodox system. However, this rarely catalyses a complete heterodox system. It is usually ad hoc, addressing individual orthodox components. Exceptions arguably include tobacco control and road safety systems, which saw incremental changes within lengthy timeframes.^102-105^ Systems responses are necessary to reduce CDoH harms.^7,25,106^ These require recognising all elements of the orthodox system (discursive and ideological, and material manifestations of these), developing a heterodox critique of dominant discursive elements, identifying interventions, and closely monitoring these for ongoing effectiveness. All of this must be supported by focused, well implemented, and clearly articulated research. This can be very effective if translated into forms readily accessible by concerned citizens. As has been demonstrated by recent revelations in relation to Australia’s casino businesses, the media has a major role to play in achieving reform. Those advocating for reform must engage regularly and consistently with media outlets. This was also a major lesson from the tobacco control movement. The avenue for articulation of heterodoxy is, arguably, the media.

 Food, alcohol, and gambling industries are powerful institutions, formed by complex relationships, powerful orthodox discourses, and (as with all institutions) largely imaginary elements dependent on orthodox discourses.^107^ All institutions are in flux, subject to change and re-conception. They are not immutable ‘structures,’ despite contrary appearances. They can be modified through application of heterodox discursive elements: the product of imaginary processes operating to critically pursue alternative institutions that are less committed to profit at all costs, and more committed to better population health and well-being.

 Successful harm prevention will necessarily involve disrupting relationships between industry, political processes, and regulation. The relationships between industry, government and regulators, and the mechanisms and institutions that perpetuate these, are a prominent quality of industry power. While this research focused on Australia, these industries are highly globalised,^108^ and previous work has also emphasised these issues.^88,106,109,110^ One key heterodox initiative is to encourage greater distance between policy and decision makers and industry, and greater integrity within political processes. This would likely require an ideological shift, and a re-definition of government roles toward protecting and promoting population health.

 Similarly, this work highlights the importance of careful interrogation of all food, alcohol, and gambling industry relationships — for researchers, PH professionals, and civil society alike. Industry funding and collaborative relationships present complex challenges. Alignment of these industries’ commercial interests with public and institutional interests requires careful consideration, to avoid inadvertently exacerbating CDoH harms.

 We need broader recognition of CDoH at policy and regulatory levels. Clear CDoH framings are essential for this.^25^ CDoH represent complex, dynamic systems shaping health at supranational, regional, national, and local levels. Heterodox discourses should focus on disrupting orthodox discourses supporting these, such as endorsements of key elements of neoliberal capitalist ideology. This is particularly so where these are normalised as ‘the way things are.’ A market society can operate with fair regulation. Profits (but perhaps not super profits) can be extracted from well-regulated market transactions.

 Within all this, PH professionals need to develop ‘political’ understandings and practice skills. In this, we can learn from and work alongside those with complementary skillsets and aligned public interests such as political scientists, investigative journalists, civil society activists, business analysts, lawyers, and others.^46,111,112^ Health, and particularly PH, has always been deeply political. However, health arguments are rarely at the forefront of major policies like industrial relations, trade, taxation, and despite piecemeal commitments to health in all policies,^113^ commitments are mostly for downstream treatment. Therefore, looking for allies and aligned causes outside of PH is highly beneficial. Notably, drawing on arguments from economics, law, business, and other areas are needed to support heterodox responses to harmful orthodox discourses. PH advances are won by continued and ongoing engagement in political processes.^114^ Advocacy is an essential role in this respect. While political access for PH personnel may not be as straightforward as some industry participants indicated in this study, organised advocacy efforts are essential and can be bolstered through well-aligned collaboration. The CDoH underpin most pressing global health challenges. Formulating PH responses addressing these will require an end to siloed PH approaches, and adoption of widespread collaborations and broader systems thinking.

###  Strengths and Limitations

 This study sought diverse perspectives from participants with experience of the food, alcohol, and gambling industries, including current and former politicians and political staffers, regulators and other public servants, industry representatives, lobbyists, journalists, and researchers. Invitations to participate received good response rates, and most invitees who declined provided a reason. Participants represented each target group and each industry, and held diverse political perspectives. These brought rich perspectives. However, this meant reaching data saturation was unlikely, and subsequently was not achieved.

 This paper seeks to elevate theoretical conceptualisations of power relevant to CDoH by framing analyses using Wood and colleagues’ *Integrated Corporate Power Framework to inform Analysis of the CDoH.*^16^ This application of a theoretical frame within an empirical CDoH study is relatively novel. Future research would benefit from incorporating similar frames in all aspects of study design and implementation.

## Conclusion

 The Australian food, alcohol, and gambling industries are powerful. Together, these have significant impacts on population health and well-being. These industries seemingly have high levels of instrumental, structural, and discursive power,^27^ with origins in material, ideational^30,101^ and relational sources. Of these, discursive power appears the most prolific. Power and its manifestations represent central aspects of the CDoH, forming seamless links to dominant systems and institutions of neoliberal capitalism, globalisation, CPA, and others. It is important to recognise this power, and take steps to reduce it to strengthen governance systems, and improve population health.

 Importantly, this requires PH advocates and researchers to adopt a political lens, with the intention of disrupting the comfortable orthodoxy that harmful commodity industries articulate, and which permeates government and regulatory systems. Population health will not be advanced with ‘business as usual.’ Business as usual is the cause of the pandemic of NCDs that now confronts the global population. Addressing this requires disruption of orthodox discourses and ideologies underpinning it.

## Ethical issues

 This project was approved by Monash University Human Research Ethics Committee, Melbourne, VIC, Australia.

## Competing interests

 Authors declare that they have no competing interests.

## Funding

 CdLV was supported by an Australian Government Research Training Program stipend and a Monash Graduate Excellence Scholarship during this project. No other project funding was received for this project.

## Supplementary files


Supplementary file 1. Sample Frame.

Supplementary file 2. Interview Guide.

## References

[1] Brownell KD, Warner KE (2009). The perils of ignoring history: Big Tobacco played dirty and millions died. How similar is Big Food? Milbank Q.

[2] Freudenberg N. Lethal but Legal: Corporations, Consumption, and Protecting Public Health. Oxford University Press; 2014.

[3] Oreskes N. Merchants of Doubt: How a Handful of Scientists Obscured the Truth on Issues from Tobacco Smoke to Global Warming. 1st ed. Bloomsbury Publishing; 2010.

[4] Petticrew M, Katikireddi SV, Knai C (2017). ‘Nothing can be done until everything is done’: the use of complexity arguments by food, beverage, alcohol and gambling industries. J Epidemiol Community Health.

[5] Capewell S, Lloyd-Williams F (2018). The role of the food industry in health: lessons from tobacco?. Br Med Bull.

[6] Lacy-Nichols J, Marten R, Crosbie E, Moodie R (2022). The public health playbook: ideas for challenging the corporate playbook. Lancet Glob Health.

[7] Knai C, Petticrew M, Mays N (2018). Systems thinking as a framework for analyzing commercial determinants of health. Milbank Q.

[8] Babor TF, Robaina K (2013). Public health, academic medicine, and the alcohol industry’s corporate social responsibility activities. Am J Public Health.

[9] Jernigan DH (2009). The global alcohol industry: an overview. Addiction.

[10] Johnson M, Livingstone C (2021). Measuring influence: an analysis of Australian gambling industry political donations and policy decisions. Addict Res Theory.

[11] Kypri K, McCambridge J, Robertson N (2019). ‘If someone donates $1000, they support you If they donate $100 000, they have bought you’ Mixed methods study of tobacco, alcohol and gambling industry donations to Australian political parties. Drug Alcohol Rev.

[12] Lacy-Nichols J, Johnson M, Cullerton K. Commercial Determinants of Health Watch: a scoping review and pilot study for systematic monitoring of lobbying and political donations. Res Sq [Preprint]. February 21, 2022. 10.21203/rs.3.rs-1367722/v1.

[13] Robertson NM, Sacks G, Miller PG (2019). The revolving door between government and the alcohol, food and gambling industries in Australia. Public Health Res Pract.

[14] Mialon M, Swinburn B, Allender S, Sacks G (2016). Systematic examination of publicly-available information reveals the diverse and extensive corporate political activity of the food industry in Australia. BMC Public Health.

[15] McKee M, Stuckler D (2018). Revisiting the corporate and commercial determinants of health. Am J Public Health.

[16] Wood B, Baker P, Sacks G (2021). Conceptualising the commercial determinants of health using a power lens: a review and synthesis of existing frameworks. Int J Health Policy Manag.

[17] Lacy-Nichols J, Marten R (2021). Power and the commercial determinants of health: ideas for a research agenda. BMJ Glob Health.

[18] Maani N, van Schalkwyk MC, Petticrew M, Buse K (2022). The pollution of health discourse and the need for effective counter-framing. BMJ.

[19] Bourdieu P. Outline of a Theory of Practice. Cambridge University Press; 1977. 10.1017/cbo9780511812507.

[20] Deer C. Doxa. In: Grenfell M, ed. Pierre Bourdieu: Key Concepts. Durham: Taylor & Francis Group; 2014:viii.

[21] Thomson P. Field. In: Grenfell M, ed. Pierre Bourdieu: Key Concepts. Durham: Taylor & Francis Group; 2014:viii.

[22] Kickbusch I, Allen L, Franz C (2016). The commercial determinants of health. Lancet Glob Health.

[23] West R, Marteau T (2013). Commentary on Casswell (2013): the commercial determinants of health. Addiction.

[24] Freudenberg N. The commercial determinants of COVID-19. In: Center on Commercial Determinants of Health. Washington, DC: Milken Institute School of Public Health, The George Washington University; 2020.

[25] de Lacy-Vawdon C, Vandenberg B, Livingstone CH (2022). Recognising the elephant in the room: the commercial determinants of health. BMJ Glob Health.

[26] Sell SK, Williams OD (2020). Health under capitalism: a global political economy of structural pathogenesis. Rev Int Polit Econ.

[27] Fuchs D (2005). Commanding heights? The strength and fragility of business power in global politics. Millennium.

[28] Foucualt M (1982). The subject and power. Crit Inq.

[29] Fuchs DA. Business Power in Global Governance. Boulder, CO: Lynne Rienner Publishers; 2007.

[30] Fuchs D, Glaab K. Material Power or Normative Conflict: Determinants of the Interaction Between Global and Local Agrifood Governance. Münster: Social Science Open Access Repository (SSOAR); 2010.

[31] Madureira Lima J, Galea S (2018). Corporate practices and health: a framework and mechanisms. Global Health.

[32] Nye J Jr. The Powers to Lead: Soft, Hard, and Smart. Cary: Oxford University Press; 2008.

[33] de Lacy-Vawdon C, Livingstone C (2020). Defining the commercial determinants of health: a systematic review. BMC Public Health.

[34] Australian Government. Lobbying Code of Conduct. 2022. https://www.ag.gov.au/system/files/2022-02/lobbying-code-of-conduct.PDF.

[35] Livingstone C, Woolley R (2007). Risky business: a few provocations on the regulation of electronic gaming machines. Int Gambl Stud.

[36] Petticrew M, Maani N, Pettigrew L, Rutter H, Van Schalkwyk MC (2020). Dark nudges and sludge in big alcohol: behavioral economics, cognitive biases, and alcohol industry corporate social responsibility. Milbank Q.

[37] Costa E, Halpern D. The Behavioural Science of Online Harm and Manipulation, and What to Do About it: An Exploratory Paper to Spark Ideas and Debate. The Behavioural Insights Team. 2019:1-82.

[38] Newall PWS (2019). Dark nudges in gambling. Addict Res Theory.

[39] Brooks R, Nguyen D, Bhatti A (2022). Use of artificial intelligence to enable dark nudges by transnational food and beverage companies: analysis of company documents. Public Health Nutr.

[40] Livingstone C, Rintoul A (2020). Moving on from responsible gambling: a new discourse is needed to prevent and minimise harm from gambling. Public Health.

[41] Francis L, Livingstone C (2021). Discourses of responsible gambling and gambling harm: observations from Victoria, Australia. Addict Res Theory.

[42] Crosbie E, Carriedo A (2022). Applying a commercial determinants of health lens to understand, expose and counter industry co-option, appeasement and partnership comment on “’Part of the solution’: food corporation strategies for regulatory capture and legitimacy. ” Int J Health Policy Manag.

[43] Crosbie E, Schmidt L (2020). Commentary on Hilton et al (2020): expanding social discourse analysis to gain traction on the broad commercial determinants of health. Addiction.

[44] Kypri K. Countering Commercial Determinants of Alcohol Harm. Paper presented at: Global Alcohol Policy Conference; October 4-6, 2017; Melbourne, Australia. https://docs.wixstatic.com/ugd/a52314_b79589e799d1483b8c480db6ef88881c.pdf. Accessed May 3, 2023.

[45] Moodie R, Stuckler D, Monteiro C (2013). Profits and pandemics: prevention of harmful effects of tobacco, alcohol, and ultra-processed food and drink industries. Lancet.

[46] Mialon M, Swinburn B, Sacks G (2015). A proposed approach to systematically identify and monitor the corporate political activity of the food industry with respect to public health using publicly available information. Obes Rev.

[47] Mialon M, Julia C, Hercberg S (2018). The policy dystopia model adapted to the food industry: the example of the Nutri-Score saga in France. World Nutr.

[48] Nyberg D (2021). Corporations, politics, and democracy: corporate political activities as political corruption. Organ Theory.

[49] Wood D, Griffiths K, Chivers C. Who’s in the Room? Access and Influence in Australian Politics. Grattan Institute; 2018.

[50] Oklobdzija S (2019). Public positions, private giving: dark money and political donors in the digital age. Res Politics.

[51] Ratcliff S, Halpin D (2021). Dark money and opaque politics: making sense of contributions to Australian political parties. Aust J Polit Sci.

[52] Milsom P, Smith R, Baker P, Walls H (2021). Corporate power and the international trade regime preventing progressive policy action on non-communicable diseases: a realist review. Health Policy Plan.

[53] Phulkerd S, Collin J, Ngqangashe Y (2022). How commercial actors used different types of power to influence policy on restricting food marketing: a qualitative study with policy actors in Thailand. BMJ Open.

[54] Kowal MS (2018). Corporate politicking, together: trade association ties, lobbying, and campaign giving. Bus Polit.

[55] Mialon M, Swinburn B, Wate J, Tukana I, Sacks G (2016). Analysis of the corporate political activity of major food industry actors in Fiji. Global Health.

[56] Maani N, Petticrew M, Galea S. The Commercial Determinants of Health. Oxford University Press; 2023.

[57] Lacy-Nichols J, Cullerton K (2023). A proposal for systematic monitoring of the commercial determinants of health: a pilot study assessing the feasibility of monitoring lobbying and political donations in Australia. Global Health.

[58] dela Rama MJ, Lester ME, Staples W (2022). The Challenges of Political Corruption in Australia, the Proposed Commonwealth Integrity Commission Bill (2020) and the Application of the APUNCAC. Laws.

[59] Parliament of Australia. National Anti-Corruption Commission Bill 2022. House of Representatives; 2022.

[60] Moodie AR (2017). What public health practitioners need to know about unhealthy industry tactics. Am J Public Health.

[61] Mialon M, Vandevijvere S, Carriedo-Lutzenkirchen A (2020). Mechanisms for addressing and managing the influence of corporations on public health policy, research and practice: a scoping review. BMJ Open.

[62] Australian Electoral Commission. Disclosure Threshold. 2022. https://www.aec.gov.au/parties_and_representatives/public_funding/threshold.htm. Accessed February 21, 2023.

[63] NSW Electoral Commission. Unlawful Political Donations. 2023. https://elections.nsw.gov.au/funding-and-disclosure/political-donations/unlawful-political-donations/prohibited-donors. Accessed February 21, 2023.

[64] Labonté R, Crosbie E, Gleeson D, McNamara C (2019). USMCA (NAFTA 20): tightening the constraints on the right to regulate for public health. Global Health.

[65] Anaf J, Baum FE, Fisher M, Harris E, Friel S (2017). Assessing the health impact of transnational corporations: a case study on McDonald’s Australia. Global Health.

[66] MacKenzie R, Mathers A, Hawkins B, Eckhardt J, Smith J (2018). The tobacco industry’s challenges to standardised packaging: a comparative analysis of issue framing in public relations campaigns in four countries. Health Policy.

[67] Madureira Lima J. Assessing power structures. In: Maani N, Petticrew M, Galea S, eds. The Commercial Determinants of Health. Oxford: Oxford University Press; 2023.

[68] Tobacco Free Portfolios. Imagine a Tobacco Free World. 2020. https://tobaccofreeportfolios.org/. Accessed February 21, 2023.

[69] The Australia Institute, Quit Nukes. Quit Nukes: The Case for Australian Superannuation Funds to be Nuclear Weapons Free. Canberra, ACT: The Australia Institute, Quit Nukes; 2021.

[70] PAX, International Campaign to Abolish Nuclear Weapons. Risky Returns: Nuclear Weapon Producers and Their Financiers. Don’t Bank on the Bomb; 2022.

[71] World Health Organization (WHO). WHO Framework Convention on Tobacco Control. Geneva, Switzerland: WHO; 2003.

[72] United Nations Office for Disarmament Affairs. Treaty on the Prohibition of Nuclear Weapons. New York: United Nations; 2017.

[73] Ulucanlar S, Fooks GJ, Gilmore AB (2016). The policy dystopia model: an interpretive analysis of tobacco industry political activity. PLoS Med.

[74] Baum FE, Margaret Anaf J (2015). Transnational corporations and health: a research agenda. Int J Health Serv.

[75] Brown SR (2019). Subjectivity in the human sciences. Psychol Rec.

[76] Godziewski C (2021). Is ‘health in all policies’ everybody’s responsibility? Discourses of multistakeholderism and the lifestyle drift phenomenon. Crit Policy Stud.

[77] Maani Hessari N, Petticrew M (2018). What does the alcohol industry mean by ‘Responsible drinking’? A comparative analysis. J Public Health (Oxf).

[78] Ramsbottom A, Petticrew M, van Schalkwyk M, Carters-White L, Benylles Y. Food as harm reduction during a drinking session: reducing the harm, or normalising harmful use of alcohol? A comparative analysis of alcohol industry and non-alcohol industry-funded advice. Res Sq [Preprint]. August 4, 2021. 10.21203/rs.3.rs-764214/v1. PMC9233813 35752850

[79] Miller HE, Thomas SL (2018). The problem with ‘responsible gambling’: impact of government and industry discourses on feelings of felt and enacted stigma in people who experience problems with gambling. Addict Res Theory.

[80] Howse E, Hankey C, Bauman A, Freeman B (2021). Are young adults’ discussions of public health nutrition policies associated with common food industry discourses? A qualitative pilot study. Aust N Z J Public Health.

[81] Farrell LC, Warin MJ, Moore VM, Street JM (2016). Emotion in obesity discourse: understanding public attitudes towards regulations for obesity prevention. Sociol Health Illn.

[82] Douglas N, Knai C, Petticrew M, Eastmure E, Durand MA, Mays N (2018). How the food, beverage and alcohol industries presented the Public Health Responsibility Deal in UK print and online media reports. Crit Public Health.

[83] Smith K, Dorfman L, Freudenberg N (2016). Tobacco, alcohol, and processed food industries - why do public health practitioners view them so differently?. Front Public Health.

[84] Huizar MI, Arena R, Laddu DR (2021). The global food syndemic: the impact of food insecurity, malnutrition and obesity on the healthspan amid the COVID-19 pandemic. Prog Cardiovasc Dis.

[85] Rossow I, Mäkelä P (2021). Public health thinking around alcohol-related harm: why does per capita consumption matter?. J Stud Alcohol Drugs.

[86] Iranpour A, Nakhaee N (2019). A review of alcohol-related harms: a recent update. Addict Health.

[87] Shield K, Manthey J, Rylett M (2020). National, regional, and global burdens of disease from 2000 to 2016 attributable to alcohol use: a comparative risk assessment study. Lancet Public Health.

[88] Lacy-Nichols J, Williams O (2021). “Part of the solution”: food corporation strategies for regulatory capture and legitimacy. Int J Health Policy Manag.

[89] Liber AC (2022). Using regulatory stances to see all the commercial determinants of health. Milbank Q.

[90] Lee K, Freudenberg N (2022). Public health roles in addressing commercial determinants of health. Annu Rev Public Health.

[91] Lencucha R, Thow AM (2020). Intersectoral policy on industries that produce unhealthy commodities: governing in a new era of the global economy?. BMJ Glob Health.

[92] McHardy J (2021). The WHO FCTC’s lessons for addressing the commercial determinants of health. Health Promot Int.

[93] Peruga A, López MJ, Martinez C, Fernández E (2021). Tobacco control policies in the 21st century: achievements and open challenges. Mol Oncol.

[94] Australian Government Department of Health and Aged Care. Tobacco Control Timeline. 2018. https://www1.health.gov.au/internet/publications/publishing.nsf/Content/tobacco-control-toc~timeline. Accessed February 21, 2023.

[95] Jarman H, Koivusalo M, Hervey TK, Young CA, Bishop LE. Research Handbook on EU Health Law and Policy. Northampton, MA: Edward Elgar Publishing; 2017.

[96] Collin J (2012). Tobacco control, global health policy and development: towards policy coherence in global governance. Tob Control.

[97] McCambridge J, Morris S (2019). Comparing alcohol with tobacco indicates that it is time to move beyond tobacco exceptionalism. Eur J Public Health.

[98] Gelius P, Messing S, Tcymbal A, Whiting S, Breda J, Abu-Omar K (2021). Policy instruments for health promotion: a comparison of WHO policy guidance for tobacco, alcohol, nutrition and physical activity. Int J Health Policy Manag.

[99] Bailey I, MacGill I, Passey R, Compston H (2012). The fall (and rise) of carbon pricing in Australia: a political strategy analysis of the carbon pollution reduction scheme. Env Polit.

[100] Hancock L. Whatever happened to the Commonweath gambling agenda? Dissent. 2012(38):55-59.

[101] Fuchs D, Glaab K (2011). Material power and normative conflict in global and local agrifood governance: the lessons of ‘Golden Rice’ in India. Food Policy.

[102] Flor LS, Reitsma MB, Gupta V, Ng M, Gakidou E (2021). The effects of tobacco control policies on global smoking prevalence. Nat Med.

[103] McDaniel PA, Smith EA, Malone RE (2016). The tobacco endgame: a qualitative review and synthesis. Tob Control.

[104] Wegman F (2017). The future of road safety: a worldwide perspective. IATSS Res.

[105] Hughes BP, Newstead S, Anund A, Shu CC, Falkmer T (2015). A review of models relevant to road safety. Accid Anal Prev.

[106] Knai C, Petticrew M, Capewell S (2021). The case for developing a cohesive systems approach to research across unhealthy commodity industries. BMJ Glob Health.

[107] Castoriadis C. The Imaginary Institution of Society. Cambridge, UK: Polity Press; 1987.

[108] Franz C, Kickbusch I (2018). The capital-NCD-nexus: the commercial determinants of health and global capital flows. Eurohealth.

[109] McCambridge J, Mialon M, Hawkins B (2018). Alcohol industry involvement in policymaking: a systematic review. Addiction.

[110] Mialon M, Swinburn B, Allender S, Sacks G (2017). ‘Maximising shareholder value’: a detailed insight into the corporate political activity of the Australian food industry. Aust N Z J Public Health.

[111] Mialon M, Anaf J, Baum F. Learning from experience: identifying key intervention points around corporate practices to improve health. In: Maani N, Petticrew M, Galea S, eds. The Commercial Determinants of Health. New York, NY: Oxford University Press; 2023:331-340.

[112] Maani N, Petticrew M, Galea S. Commercial determinants of health: a research and translational agenda. In: Maani N, Petticrew M, Galea S, eds. The Commercial Determinants of Health. New York, NY: Oxford University Press; 2023:353-357.

[113] Smith J, Griffiths K, Judd J (2018). Ten years on from the World Health Organization Commission of Social Determinants of Health: progress or procrastination?. Health Promot J Austr.

[114] Hunter EL (2016). Politics and public health-engaging the third rail. J Public Health Manag Pract.

