# Supplementary material for: Power and Other Commercial Determinants of Health: An Empirical Study of the Australian Food, Alcohol, and Gambling Industries
Source: Int J Health Policy Manag. 2023 May 28;12:7723. doi: 10.34172/ijhpm.2023.7723 (PMC10461899; doi:10.34172/ijhpm.2023.7723)
Supplement: Supplementary file 1 — Sample Frame. [file ijhpm-12-7723-s001.pdf]

Article title: Power and Other Commercial Determinants of Health: An Empirical Study of the Australian Food, Alcohol, and Gambling Industries

Journal name: International Journal of Health Policy and Management (IJHPM)

Authors' information: Cassandra de Lacy-Vawdon[1,2]*, Brian Vandenberg[3], Charles Livingstone[2]

[1]Department of Public Health, School of Psychology and Public Health, La Trobe University, Melbourne, VIC, Australia.

[2]School of Public Health and Preventive Medicine, Monash University, Melbourne, VIC, Australia.

[3]School of Social Sciences, Monash University, Melbourne, VIC, Australia.

(*Corresponding author: c.delacy-vawdon@latrobe.edu.au)

Citation: de Lacy-Vawdon C, Vandenberg B, Livingstone C. Power and other commercial determinants of health: an empirical study of the Australian food, alcohol, and gambling industries. Int J Health Policy Manag. 2023;12:7723. doi: 10.34172/ijhpm.2023.7723

Supplementary file 1. Sample Frame

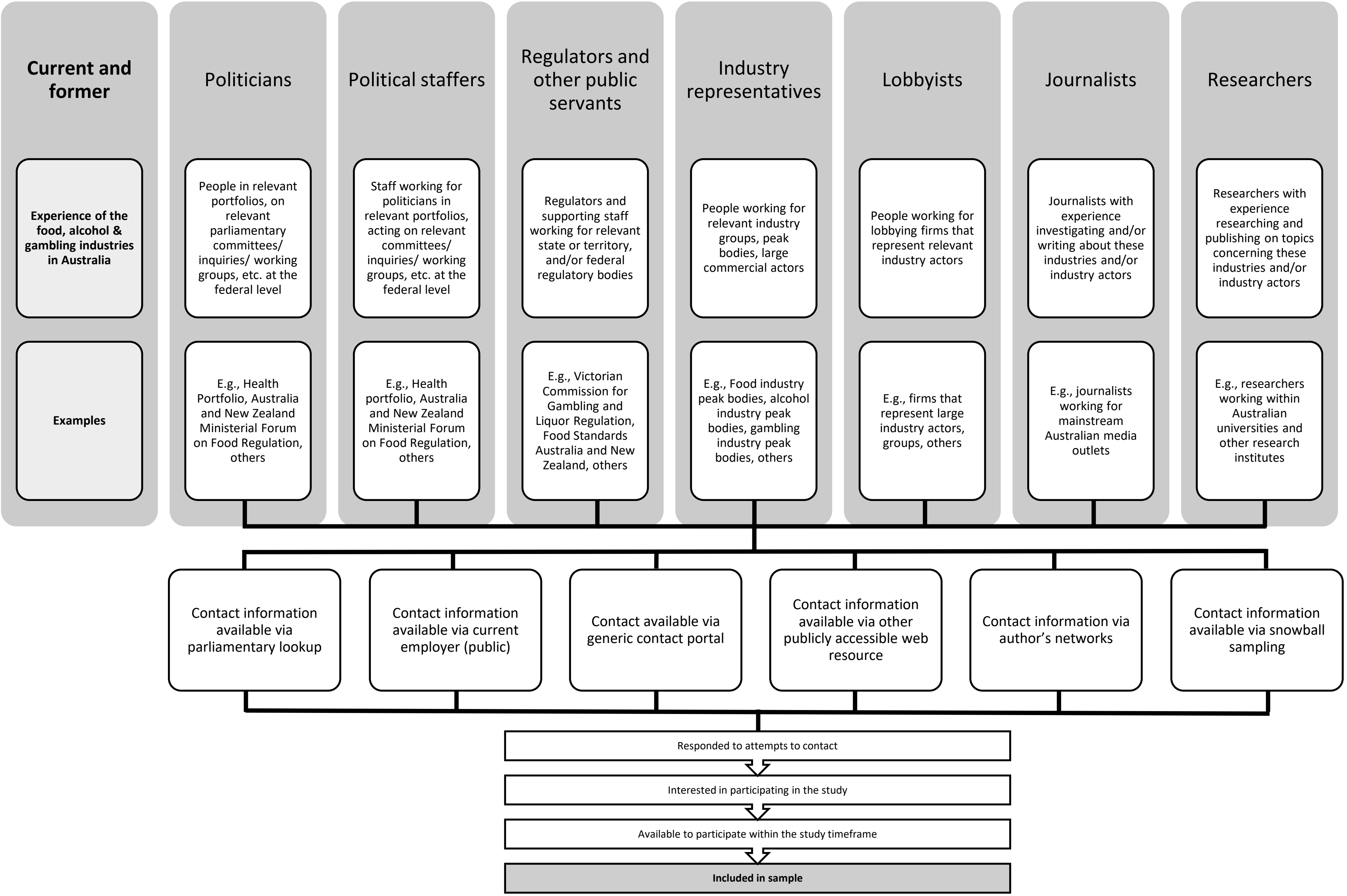
Current and former
Politicians
Political staffers
Regulators and other public servants
Industry representatives
Lobbyists
Journalists
Researchers
Experience of the food, alcohol & gambling industries in Australia
People in relevant portfolios, on relevant parliamentary committees/ inquiries/ working groups, etc. at the federal level
Staff working for politicians in relevant portfolios, acting on relevant committees/ inquiries/ working groups, etc. at the federal level
Regulators and supporting staff working for relevant state or territory, and/or federal regulatory bodies
People working for relevant industry groups, peak bodies, large commercial actors
People working for lobbying firms that represent relevant industry actors
Journalists with experience investigating and/or writing about these industries and/or industry actors
Researchers with experience researching and publishing on topics concerning these industries and/or industry actors
Examples
E.g., Health Portfolio, Australia and New Zealand Ministerial Forum on Food Regulation, others
E.g., Health portfolio, Australia and New Zealand Ministerial Forum on Food Regulation, others
E.g., Victorian Commission for Gambling and Liquor Regulation, Food Standards Australia and New Zealand, others
E.g., Food industry peak bodies, alcohol industry peak bodies, gambling industry peak bodies, others
E.g., firms that represent large industry actors, groups, others
E.g., journalists working for mainstream Australian media outlets
E.g., researchers working within Australian universities and other research institutes
Contact information available via parliamentary lookup
Contact information available via current employer (public)
Contact available via generic contact portal
Contact information available via other publicly accessible web resource
Contact information via author's networks
Contact information available via snowball sampling
Responded to attempts to contact
Interested in participating in the study
Available to participate within the study timeframe
Included in sample
