# Supplementary material for: Power and Other Commercial Determinants of Health: An Empirical Study of the Australian Food, Alcohol, and Gambling Industries
Source: Int J Health Policy Manag. 2023 May 28;12:7723. doi: 10.34172/ijhpm.2023.7723 (PMC10461899; doi:10.34172/ijhpm.2023.7723)
Supplement: Supplementary file 2 — Interview Guide. [file ijhpm-12-7723-s002.pdf]

Article title: Power and Other Commercial Determinants of Health: An Empirical Study of the Australian Food, Alcohol, and Gambling Industries

Journal name: International Journal of Health Policy and Management (IJHPM)

Authors' information: Cassandra de Lacy-Vawdon[1,2]*, Brian Vandenberg[3], Charles Livingstone[2]

[1]Department of Public Health, School of Psychology and Public Health, La Trobe University, Melbourne, VIC, Australia.

[2]School of Public Health and Preventive Medicine, Monash University, Melbourne, VIC, Australia.

[3]School of Social Sciences, Monash University, Melbourne, VIC, Australia.

(*Corresponding author: c.delacy-vawdon@latrobe.edu.au)

Citation: de Lacy-Vawdon C, Vandenberg B, Livingstone C. Power and other commercial determinants of health: an empirical study of the Australian food, alcohol, and gambling industries. Int J Health Policy Manag. 2023;12:7723. doi: 10.34172/ijhpm.2023.7723

Supplementary file 2. Interview Guide

The interview guide is as follows. Note this is a guide and not an indication of questions that will be asked verbatim.

**Introduction**<br>• Brief introduction<br>• Check received Explanatory Statement<br>• Check verbal consent via phone/Zoom
**Begin recording**
**Background**<br>• What is your current role?<br>• What is your primary area of focus?<br>• Could you briefly describe previous roles have you held that have provided insight into the food, alcohol and/or gambling industries in Australia?<br>• Can you briefly describe your knowledge of and/or experience with the Australian food, alcohol, and gambling industries?
**Perceptions of the food/alcohol/gambling industries**<br>• Briefly, how would you describe the food/alcohol/gambling industries in Australia?<br>○ Please provide a couple of sentences on overall perceptions

- What are some of your perceptions of the integration of the food/alcohol/gambling industries in Australia?
  - Within each industry (vertical integration)
  - Between industries (horizontal integration)
- How would you describe the relationships between these industries?
  - People
  - Culture
  - Goals/objectives/activities
  - Legal/regulatory structures
- What would you describe as some of the similarities between these industries?
- What would you describe as some of the points of difference between these industries?
  - How to they try to differentiate themselves from other industries?
- How would you say these industries affect the health and wellbeing of the population?

Perceptions of food/alcohol/gambling industry power

Within this work we are also interested in exploring the perceived power of the food/alcohol/gambling industries in Australia. Notably, power often falls within categories of hard power (coercive power, including legal and/or economic threats, use of force, etc.) and soft power (persuasive power, shaping preferences, influencing through perceived legitimate or moral authority, etc.).

- How would you describe the power held by the food/alcohol/gambling industries?
  - Social power
  - Political influence
  - Regulatory
  - Financial
- Do you have any particular examples of hard (coercive) power?
- Do you have any particular examples of soft (persuasive) power? – including public perception, often unseen influence
- Where do you think this power come from? How is it reinforced? – i.e. systems of power
- What does this power do/achieve?

Perceptions of food/alcohol/gambling industry external relationships

- How do you perceive the relationships between the food/alcohol/gambling industries and key personnel within
  - Government personnel
    - Ministers
    - senators
    - advisors
    - public servants
  - Advisory bodies
  - Lobbying
  - Consultation
  - and other roles (i.e., corporate and political connections).
- How are these relationships carried out in formal settings?
- How are these relationships carried out informally?
- What facilitates/promotes these relationships?
- What do these relationships achieve for industry?
- What are the implications of these relationships for policy/regulation?
- What are the implications of these relationships for public health and wellbeing?

Perceptions of food/alcohol/gambling industry influence (i.e., what they do)

• Do you think the food/alcohol/gambling industries have influence over public health and public policy decisions?<br>○ How much influence do they have?<br>• What are these influences?<br>• How do these come about?<br>• Do you have any particular examples?<br>• What are the outcomes of these influences? (from your perspective)
**Perceptions of mechanisms/relationships reinforcing food/alcohol/gambling industry influence (i.e., how they do it)**<br>• What influence does the food/alcohol/gambling industry have over public policy and public health?<br>• How is this the case?<br>• What are the mechanisms that reinforce this?<br>○ Regulatory, legal, social, political, commercial, etc.<br>• What role do relationships have in this influence?<br>• Can you describe any particular examples of this?<br>• What are the outcomes of this for public health and wellbeing? (from your perspective)
**Perceptions of opportunities and needs for intervention**<br>• Do you see any opportunities or needs are there for intervention? (from your perspective)<br>○ What should be done?<br>• What do you perceive as opportunities for intervention to reduce the influence of the food/alcohol/gambling industries in public policy and public health?<br>○ What can be done?<br>• What should be some key features of these interventions?<br>• What is working well that should be enhanced? How?<br>• What could be the consequences if these are not addressed?<br>○ For policy, regulation, public health, etc.
**Priorities for intervention(s)**<br>• What do you think are the most important areas of intervention in order to minimise or prevent harm and maximise benefits to public health?<br>• Which intervention(s) would be most important to implement? Why?<br>○ Timely<br>○ Cost-benefit<br>○ Simplicity<br>○ Addressing systems of harm<br>○ Trigger points within systems<br>○ Addressing multiple harms at once<br>• What would you prioritise first? Why?<br>• Which intervention(s) could have the greatest impact?
**Thank you**
